# Protective Effect of Glycyrrhizin, a Direct HMGB1 Inhibitor, on Focal Cerebral Ischemia/Reperfusion-Induced Inflammation, Oxidative Stress, and Apoptosis in Rats

**DOI:** 10.1371/journal.pone.0089450

**Published:** 2014-03-04

**Authors:** Gu Gong, Lei Xiang, Libang Yuan, Ling Hu, Wei Wu, Lin Cai, Liang Yin, Hailong Dong

**Affiliations:** 1 Department of Anaesthesiology, General Hospital of the People's Liberation Army, Chengdu, Sichuan, China; 2 Department of Neurology, Tianjin Huanhu Hospital, Tianjin, China; 3 Department of Anaesthesiology, Xijing Hospital, Fourth Military Medical University, Xi'an, Shaanxi, China; University of Missouri, United States of America

## Abstract

**Aim:**

Gl**y**cyrrhizin (GL) has been reported to protect against ischemia and reperfusion (I/R)-induced injury by inhibiting the cytokine activity of high mobility group box 1 (HMGB1). In the present study, the protective effects of GL against I/R injury, as well as the related molecular mechanisms, were investigated in rat brains.

**Methods:**

Focal cerebral I/R injury was induced by intraluminal filamentous occlusion of the middle cerebral artery (MCA) in Male Sprague-Dawley rats. GL alone or GL and rHMGB1 were administered intravenously at the time of reperfusion. Serum levels of HMGB1 and inflammatory mediators were quantified via enzyme-linked immunosorbent assay (ELISA). Histopathological examination, immunofluorescence, RT-PCR and western blotting analyses were performed to investigate the protective and anti-apoptotic effects and related molecular mechanisms of GL against I/R injury in rat brains.

**Results:**

Pre-treatment with GL significantly reduced infarct volume and improved the accompanying neurological deficits in locomotor function. The release of HMGB1 from the cerebral cortex into the serum was inhibited by GL administration. Moreover, pre-treatment with GL alleviated apoptotic injury resulting from cerebral I/R through the inhibition of cytochrome C release and caspase 3 activity. The expression levels of inflammation- and oxidative stress-related molecules including TNF-α, iNOS, IL-1β, and IL-6, which were over-expressed in I/R, were decreased by GL. P38 and P-JNK signalling were involved in this process. All of the protective effects of GL could be reversed by rHMGB1 administration.

**Conclusions:**

GL has a protective effect on ischemia-reperfusion injury in rat brains through the inhibition of inflammation, oxidative stress and apoptotic injury by antagonising the cytokine activity of HMGB1.

## Introduction

Ischemic stroke remains one of the leading cause of death and disability worldwide. Recent insight into the basic mechanism involved in ischemic stroke indicates that endothelial dysfunctions along with oxidative stress and neuroinflammation represent key elements in the occurrence and development of ischemic brain damage that results in cell damage and death [1], [2]. Within hours of the ischemic insult, infiltrating leukocytes, as well as resident brain cells including neurons and glia, may release pro-inflammatory mediators such as cytokines, chemokines, and oxygen/nitrogen free radicals that contribute to the evolution of tissue damage [3]. Furthermore, the cerebral ischemia that occurs in brain cells affected by a stroke triggers a complex array of molecular and cellular alterations including the activation of signalling pathways that may either contribute to neuronal damage or protect neurons. Mitogen-activated protein kinases (MAPKs) have crucial roles in signal transduction from the cell surface to the nucleus and regulate cell death and survival processes. Among the MAPK pathways known to be activated in neurons in response to ischemia are the JNK, ERK, and p38 MAPK pathways [4], [5].

High mobility group box 1 (HMGB1), a ubiquitous and abundant nuclear protein, can either be passively released into the extracellular milieu in response to necrotic signals or actively secreted in response to inflammatory signals [6]–[8]. Recently, HMGB1 has been reported to be a potent pro-inflammatory cytokine-like factor that contributes to the pathogenesis of vasculature and connects excitotoxicity-induced acute damage processes with delayed inflammatory processes in the post-ischemic brain [9], [10]. The receptor for advanced glycation end products (RAGE), one of the most important receptors for HMGB1, functions as a sensor of necrotic cell death, and the HMGB1–RAGE signalling axis contributes to inflammation and ischemic brain damage [11]. Intravenous injection of neutralising anti-HMGB1 mAb or intranasal delivery of HMGB1 siRNA conferred robust neuroprotection in the post-ischemic brain by antagonising the pro-inflammatory function of HMGB1 [12], [13].

Glycyrrhizin (GL) is a major active constituent of *Glycyrrhiza glabra* root and is composed of a molecule of glycyrrhizic acid and two molecules of glucuronic acid. This compound has been associated with numerous pharmacological effects, including anti-inflammatory, anti-viral, anti-tumour, and hepatoprotective activities, and is commonly used in Asia to treat patients with chronic hepatitis [14]–[17]. It was reported by Sitia et al [18] that, as an HMGB1 inhibitor, GL binds directly to HMGB1 (Kd ∼150 µM), interacting with two shallow concave surfaces formed by the two arms of both HMG boxes. GL has been reported to protect from I/R-induced injury in many organs, including the liver [19], spinal cord [20] and heart [21], by inhibiting the chemoattractant and mitogenic functions of HMGB1. Recently, a robust neuroprotective effect of Stronger Neo-Minophagen C (SNMC), a GL-containing preparation, has been reported in the post-ischemic brain, and this neuroprotective effect is due, at least in part, to an anti-inflammatory effect [22]. However, it is not known if the neuroprotective effect of GL occurs through the antagonism of HMGB1 and the ensuing molecular signalling events. Therefore, the aim of this study was to investigate the potential protective effect of GL, as well as the related mechanisms, against I/R injury in the rat brain, mainly in relation to the following aspects: (1) the neuroprotective effects of GL on focal cerebral ischemia; (2) the release of HMGB1 in rat serum and brain; (3) the effect of GL on the alleviation of apoptosis caused by I/R injury; (4) the expression of HMGB1-dependent inflammation- and oxidative stress-related molecules; (5) the involvement of certain MAPK pathways that are modulated by GL.

## Materials and Methods

### Animals and groups

All experiments were performed in accordance with the “Guide for the Care and Use of Laboratory Animals” published by the US NIH (National Institutes of Health Publication No. 85-23, revised 1996) and were approved by the Committee on Animal Experiments of the Sichuan Neurosurgical Institute. Male Sprague-Dawley rats weighing 325±25 g were obtained from the Experimental Animal Centre of the Sichuan Neurosurgical Institute (China) and were allowed free access to laboratory chow and tap water in day-night quarters at 25°C. Rats were randomly divided into the following four experimental groups of 8 animals each: (1) healthy control, sham-operated rats (the sham group); (2) I/R rats pre-treated with saline (the NS group); (3,4,5) I/R rats pre-treated with GL separately at doses of 2 mg/kg, 4 mg/kg and 10 mg/kg (the 2 mg/kg, 4 mg/kg and 10 mg/kg GL groups); (6) I/R rats pre-treated with GL and recombinant HMGB1 (100 µg per rat) (the GL+rHMGB1 group). In the pre-treatment groups, after 30 min ischemia and before reperfusion, NS, GL or GL plus rHMGB1 was administered intravenously in the tail vein in a volume of 0.5 ml followed by a 48 h reperfusion.

### Focal cerebral I/R

Focal cerebral ischemia was induced by performing intraluminal filamentous occlusion of the middle cerebral artery (MCA) for 60 min, according to methods that have been described previously [23]. After 60 min of MCA occlusion, reperfusion of the MCA was initiated by removing the MCA-occlusive filament. The right femoral artery was cannulated to monitor mean arterial blood pressure, arterial blood gases, and pH. Regional cerebral blood flow was monitored using a laser Doppler flowmeter (Periflux System 5000; Perimed, Jarfalla, Sweden). A thermoregulated heating pad and an overhead heating lamp were used to maintain a rectal temperature of 37±0.5°C. In sham-operated rats, an incision was made over the MCA, but the artery was not occluded. The cerebral I/R injury model was developed with 60 min of MCA occlusion followed by 48 h reperfusion.

### Triphenyltetrazolium chloride (TTC) staining and infarct volume assessments

Coronal brain sections (2-mm thickness) were incubated with 2% TTC at 37°C for 30 min with gentle shaking and then fixed with 10% formalin in PBS. The stained slices were photographed, and the size of the infarct was quantified using NIH image software.

TTC staining was used to measure infarction volumes two days after I/R (n = 8 per group) and whole brains were dissected coronally into 2-mm brain slices. Two investigators blinded to the study protocol measured the infarct sizes with a computerised image analyser. To account for cerebral oedema and differential shrinkage resulting from tissue processing, the areas of ischemic lesions were determined by subtracting the areas in ipsilateral hemispheres from those of contralateral hemispheres. Infarct volumes were calculated (in mm^3^) by multiplying the summed section infarct areas by the section thickness.

### Evaluation of neurological deficits

Neurological deficits were evaluated by two methods. In the rota-rod test, rats were conditioned for 3 days before MCA occlusion on an accelerating rota-rod cylinder at 5 to 15 rpm. Rats that could stay on the rotating rod at 15 rpm for 180 s were subjected to MCA occlusion. After MCA occlusion, each rat was subjected to trials conducted at 3 different speeds (5, 10 and 15 rpm), and the mean duration of three trials at each speed on the rota-rod was recorded. The other test was a neurological scoring method, which was performed essentially as described by Bederson et al [24]. The scores were categorised according to four grades (0, normal; 1, moderate; 2, considerable; 3, severe). The modified Neurological Severity Scores system consists of motor, sensory, balance, and reflex tests, all of which are graded using a scale of 0–18 (normal: 0, maximal deficit: 18). The neurological evaluation was performed by an investigator who was blind to the treatment condition.

### Serum ELISA detection

Blood samples (0.5 ml) were collected from the femoral vein at 6, 12, 24 and 48 h after reperfusion. Serum was isolated from the blood after centrifugation at 14 000 rpm for 20 min at 4°C. After centrifugation, serum was frozen at −80°C until enzyme-linked immunosorbent assay (ELISA) analyses were performed. HMGB1 concentrations and the levels of inflammatory mediators (TNF-α, iNOS,IL-1β, COX-2 and IL-6) in the serum samples were quantified using specific ELISA kits for rats according to the manufacturer's instructions (Biosource International Inc., Camarillo, CA, USA).

### Western blot

Total protein extracts or detached subcellular fractions from rat cerebral tissue were prepared as described previously [25]. The antibodies and the dilutions were as follows: p-JNK No. 9255 (1∶2000), t-JNK No. 9252 (1∶1000), p-ERK [1/2] No. 9101 (1∶1000), t-ERK [1/2] No. 4695 (1∶1000), p-p38 No. 9211(1∶1000), t-p38 No. 9212 (1∶1000) (Cell Signaling Technology, Danvers, Mass), Cytochrome c (1 µg/ml, No. ab90529, Abcam Plc., Cambridge, UK) and HMGB1 (1 µg/ml, No. ab18256, Abcam Plc., Cambridge, UK). Horseradish peroxidase–coupled rabbit and mouse IgG (1∶2000) were used as secondary antibodies. Proteins were separated on 10–20% SDS polyacrylamide gels and electrophoretically transferred to polyvinylidene difluoride membranes, which were then incubated overnight at 4°C with the primary antibody diluted in blocking solution. After washing, membranes were treated with horseradish peroxidase-conjugated secondary antibodies and then with enhanced chemiluminescence reagent (Amersham Biosciences, Piscataway, NJ). The film was scanned with a GS-700 imaging densitometer (Bio-Rad Laboratories, Hercules, CA), and the results were quantified with Multi-Analyst software (Bio-Rad Laboratories).

### RNA preparation and reverse transcription-PCR

Total RNA was prepared using Trizol reagent (Invitrogen, Gaithersburg, MD) according to the manufacturer's instructions, and 1 µg RNA samples were used for cDNA synthesis. First-strand cDNA synthesis was primed with random hexamers and conducted according to the manufacturer's specifications (RT-PCR kit; Roche, Mannheim, Germany). cDNA equivalent to 200 ng of total RNA was subjected to PCR using the manufacturer's protocol (PCR core kit; Roche). The sense and antisense primers used for the analysis of rat HMGB1, TNF-α, IL-1β, iNOS, COX-2, IL-6 and GAPDH expression were as follows. HMGB1: 5′-CTGATGCAGCTTA TACGAAG-3′ and 5′-TCAGGTAAGGAGCAGAACAT- 3′ (460 bp). TNF-α: 5′-CCC CTTTATCGTCTACTCCTC-3′ and 5′-GCTGGTAGTTTAGCTCCGTTT-3′ (553 bp). IL-1β: 5′-TCATTGTGGCTGTGGAGAAG-3′ and 5′-CTATGTCCCGACCATTGCTG-3′ (579 bp). iNOS: 5′-GCATCCCAAGTACGAGTGGT-3′ and 5′-GAAGGCGTAG CTGAACAAGG-3′ (700 bp). COX-2: 5′-GTGGGATGACGAGCGACTGT-3′ and 5′ -TTTCAGGGAGAAGCGTTTGC-3′ (454 bp). IL-6: 5′-GGATACCACCCACAACAGAC-3′ and 5′-TTGCCGAGTAGACCTCATAG-3′ (520 bp). GAPDH: 5′-CCATCAC TGCCACTCAGAAGA-3′ and 5′-CATGAGGTCCACCACCCTGT- 3′ (446 bp). The annealing temperature was 55°C for all primer pairs.

### Immunofluorescence and TUNEL staining

Brains were isolated and fixed with 4% paraformaldehyde by transcardial perfusion and post-fixed in the same solution overnight at 4°C. Coronal brain sections (20 µm) from the ischemic core region were prepared using a vibratome (Leica, Solms, Germany), and immunological staining was performed using a previously described floating method [26]. Rabbit anti-HMGB1 (ab18956; Abcam, Cambridge, UK) antibody was used at a 1∶500 dilution. FITC-labelled anti-rabbit IgG (Jackson ImmunoResearch Laboratories, West Grove, PA) was used as a secondary antibody for anti-HMGB1.

The terminal deoxynucleotidyl transferase-mediated dUTP-biotin nick end labelling (TUNEL) assay was carried out with a commercial TUNEL kit (Roche, Switzerland) according to the manufacturer's instructions. Apoptotic cells in the rat heart after I/R in each group were counted separately using Image J software.

### Analysis of superoxide and peroxynitrite formation

The oxidative fluorescent dye dihydroethidium (DHE; Sigma-Aldrich) was used to evaluate in situ superoxide production [27]. Frozen, enzymatically intact brains from the ischemic core region were cut into 30-µm thick sections and placed on glass slides. The sections were simultaneously incubated with DHE (10 µmol/L) in phosphate-buffered saline for 30 min at 37°C, in a humidified chamber that was shielded from light. We also monitored peroxynitrite formation by detecting nitrosylated tyrosine residues on proteins. We performed immunostaining with an anti-3-nitrotyrosine (3-NT) antibody (1∶1000; Upstate Biotechnology). Coronal sections cut through the striatum (3 sections per brain, 1 mm in width) were imaged in parallel. Superoxide or 3-NT–positive cells were manually counted in the eight regions around the striatum under ×100 magnification using a laser scanning confocal microscope equipped with a Bio-Rad MRC 1024 (argon and krypton).

### Measurement of lipid peroxidation

Malondialdehyde (MDA) was estimated as an indicator of lipid peroxidation (n = 8 per group). Brain tissues were homogenised with sodium phosphate buffer (pH 7.4). The reagents (1.5 ml acetic acid, 1.5 ml thiobarbituric acid, and 0.2 ml sodium dodecyl sulphate) were added to 0.1 ml of processed tissue sample. The mixture was then heated at 100°C for 60 min. The mixture was cooled with tap water and 5 ml of n-butanol∶pyridine (15∶1), and 1 ml of distilled water was added. After centrifugation at 4000 rpm for 10 min, the organic layer was withdrawn and the absorbance was measured at 532 nm using a spectrophotometer.

### Statistical analysis

All data were expressed as the means±SEM (standard error of the mean). SPSS 17.0 was used for statistical analysis of the data. The concentrations of serum HMGB1 and inflammatory mediators were analysed using two-way repeated measures (time and group) analysis of variance followed by the post hoc Student-Newman-Keuls test. The significance level was set at p<0.05.

## Results

### Neuroprotective effects of GL on focal cerebral ischemia

**Figure 1:**
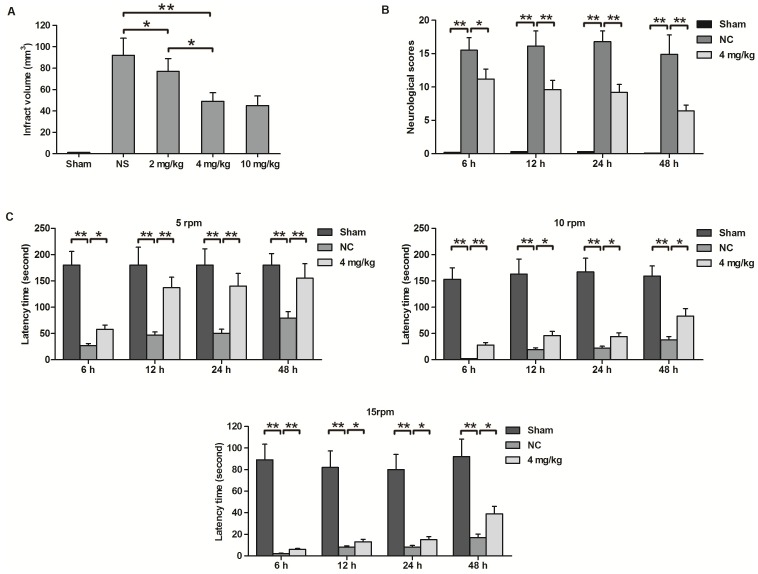
The neuroprotective effect of GL against cerebral I/R injury in rats. A) Cerebral infarction induced by MCA occlusion was evaluated 48 h after reperfusion by TTC staining of brain slices from rats treated with different doses of GL or NS. Sham-operated rats showed no infarction or TTC staining. The infarct volumes were quantified using computerised image analysis. B) Neurological scoring was carried out according to the categories described in the Materials and Methods. C) Neurological deficits in rats after MCA occlusion were examined using the rota-rod test. In the rota-rod test, trials were performed at 3 different speeds, and the time intervals running on the rod were determined for each rat after reperfusion. Values are means±SEM, n = 8 for each group. *P<0.05, **P<0.01 (t test).

No damage was observed upon TTC staining in the cerebrums of sham-group rats, whereas MCA occlusion for 60 min in rats produced massive infarction 48 h after reperfusion. Pre-treatment with different concentrations of GL significantly reduced the infarct volumes in a dose-dependent manner in focal cerebral I/R rats. There were no significant differences between the infarct volumes of the 4 mg/kg and 10 mg/kg GL-pre-treatment groups. Hence, a dose of 4 mg/kg of GL was selected for subsequent experiments, considering the potential for drug toxicity (Figure 1A).

High Neurological Severity Scores in the NS rats persisted for up to 48 h. Consistent with the reduced infarct volumes, when 4 mg/kg GL was administered at 30 min after ischemia and before reperfusion, the mean modified Neurological Severity Scores were significantly lower than those of rats in the PBS-treated NS group from 6 h to 48 h. From 6 h to 24 h, the Neurological Severity Scores declined slowly and reached the minimum value at 48 h after reperfusion in the GL group (Figure 1B). Motor activities were assessed using the rota-rod test at 5, 10 or 15 rpm speed loads. Pre-treatment with 4 mg/kg GL markedly improved the neurological deficits observed on the rota-rod test from 6 h to 48 h, regardless of whether the 5, 10 or 15 rpm speed load was used (Figure 1C). These results showed that pre-treatment with GL had neuroprotective effects on the post-ischemic brain, which were manifested as improvements in motor impairments and neurological deficits scores.

### Inhibition of HMGB1 release by GL in rats that underwent MCAO

**Figure 2:**
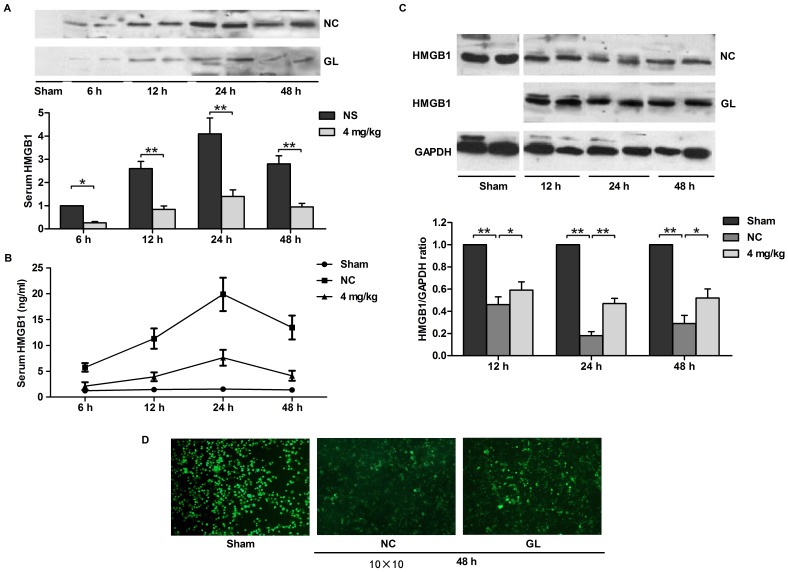
Inhibition of HMGB1 release by GL in the I/R brain. A) The serum HMGB1 concentrations of 4 mg/kg GL-pre-treated rats were determined by immunoblot at the indicated times after 1 h of MCAO. B) Serum HMGB1 concentrations were also determined by ELISA. Values are means±SEM, n = 8. **P<0.01 (t test). C) HMGB1 levels in I/R hemispheres were determined by immunoblot at various times after 1 h of MCAO. GAPDH was used as a loading control. D) Immunofluorescence staining showing different extracellular and intracellular distributions of HMGB1 in the I/R hemispheres after 48 h reperfusion. Values are means±SEM, n = 8 for each group. *P<0.05, **P<0.01 (t test).

We observed that plasma HMGB1 levels rapidly increased starting 6 h after MCAO for 1 h, peaked at 24 h after reperfusion, and then declined slowly (Figure 2A–B). When rats were pre-treated with 4 mg/kg GL, the plasma HMGB1 level was significantly decreased compared with that observed in the NS group at any time during the experimental procedure (Figure 2A–B). At 12 h after MCA occlusion (MCAO)/reperfusion, brain HMGB1 levels in the infarct area declined significantly to below the basal level and reached a minimum value at 24 h after MCAO/reperfusion. Moreover, at each time point from 12 h to 48 h after MCAO/reperfusion, brain HMGB1 levels after treatment with GL were significantly higher than those observed in the NS group (Figure 2C).

Immunofluorescence staining of nuclear HMGB1 was observed in the cerebral cortex in rats from the sham group (Figure 2D). However, the staining almost disappeared in the core of the infarct in the NS group 48 h after reperfusion, and markedly increased HMGB1 staining was observed in the extracellular space. The number of nuclear HMGB1-positive cells significantly increased upon pre-treatment with 4 mg/kg GL, and this was accompanied by a decrease in extracellular HMGB1 staining. These data suggested that increased serum levels of HMGB1 are probably attributable to spill-over from necrotic neural cells during cerebral ischemia and that GL may have a protective effect against MCAO/reperfusion-mediated cell death in neural cells, thereby decreasing the amount of HMGB1 released from the cerebral cortex into the serum.

### GL inhibits HMGB1-dependent apoptotic injury

**Figure 3:**
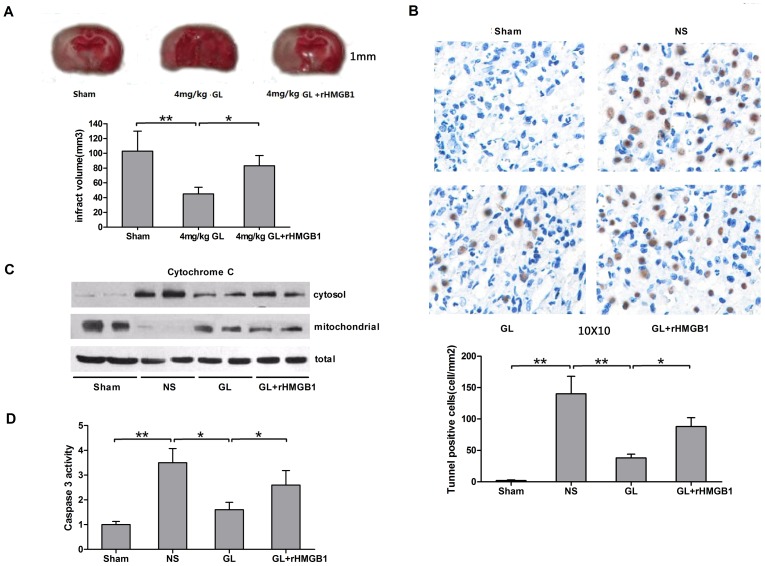
The protective effect of GL on I/R-induced infarct volume and apoptosis injury is HMGB1 dependent. A) Brain infarction induced by right MCA occlusion was evaluated by TTC staining from rats pre-treated with 4 mg/kg GL with or without 100 µg recombinant HMGB1, and the infarct volumes 48 h after reperfusion were quantified using NIH image software. B) Representative photomicrographs show TUNEL staining for apoptotic cells in rat brains at 48 h after reperfusion in the sham, NS, GL and GL+rHMGB1 groups. Effects on the severity of cerebral apoptosis are shown in an average quantitative analysis of the number of TUNEL-positive cells. C) Representative blots showing the effects of GL with or without rHMGB1 treatment on Cytochrome c translocation between the mitochondrial and cytosolic fractions. D) Effects of GL with or without rHMGB1 treatment on caspase-3 activity in areas at the risk zone of cerebral tissues at 48 h after reperfusion. Values are means±SEM, n = 8 for each group. *P<0.05, **P<0.01 (t test).

To further demonstrate that the protective effect of GL against brain I/R injury occurs via antagonism of HMGB1 function, we determined the infarct volumes of rat brains under conditions in which rHMGB1 was included. As shown in Figure 3A, although the infarct volume was significantly decreased in the 4 mg/kg GL group compared with the NS group, co-treatment with 4 mg/kg GL and 100 µg rHMGB1 30 min before ischemia significantly alleviated these changes, resulting in infarct volumes similar those observed in the NS group.

Next, we investigated whether the anti-inflammatory effect of GL can alleviate I/R-induced, HMGB1-dependent apoptosis and whether cytochrome C and caspase 3, the two most important players, are involved in this process. As depicted in Figure 3B, a large number of TUNEL-positive cells was observed in the right cortex of rats subjected to I/R injury, whereas TUNEL positive cells were not detected in the right cortex of sham-operated rats. Upon pre-treatment with 4 mg/kg GL, the number of TUNEL-positive cells was significantly reduced in the right cortex compared with the NS group. Moreover, co-treatment with rHMGB1 increased the number of TUNEL-positive cells, as expected.

In the sham group, under basal conditions, cytochrome C was predominantly expressed in the mitochondrial fraction (Figure 3C). The NS group exhibited a significant decrease in cytochrome C expression in the mitochondrial fraction when compared with the sham group, whereas an increase was observed in the cytosolic fraction. Pre-treatment with GL resulted in a significant increase in cytochrome C expression in the mitochondrial fraction and a decrease in the cytosolic fraction compared with the NS group. However, expression still did not reach the level observed in the sham group. Moreover, co-treatment with rHMGB1 can partly abolish the effect of GL on the subcellular distribution of cytochrome C proteins between the cytosolic and mitochondrial fractions. There was no significant difference in the total expression of cytochrome C protein between each group (Figure 3C). As an executioner caspase, the change in caspase 3 activity was similar to that observed with cytochrome C expression in the cytosolic fraction (Figure 3D). These results demonstrate that the administration of GL significantly alleviates the cerebral cell apoptotic injury caused by I/R through the antagonism of HMGB1 function and that the mechanism involved regulation of cytochrome C release and caspase 3 activation.

### GL inhibits HMGB1-dependent inflammatory molecule expression and oxidative stress

**Figure 4:**
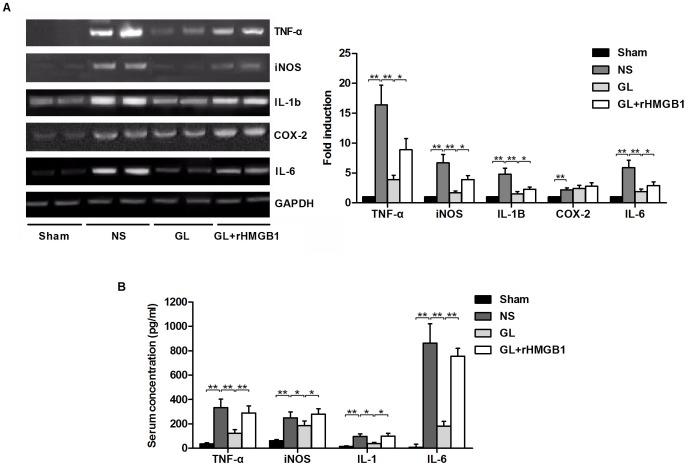
Expression of inflammation- and oxidative stress-related molecules in the brain and serum of MCA-occluded rats at 48 h after reperfusion. A) Representative blots showing the effects of GL with or without rHMGB1 treatment on mRNA expression levels of pro-inflammatory and oxidative stress markers: TNF-α, iNOS, IL-1β, COX-2 and IL-6. GAPDH was used as a loading control. The bar graph showing semi-quantitative densitometric analysis summarises the fold change of TNF-α, iNOS, IL-1β, COX-2 and IL-6 expression in each group. B) Serum concentrations of TNF-α, iNOS, IL-1β, COX-2 and IL-6 at 48 h after I/R in each groups are determined. Values are means±SEM, n = 8 for each group. *P<0.05, **P<0.01 (t test).

The expression of mRNA encoding inflammation- and oxidative stress-related molecules was determined by RT-PCR. As showed in Figure 4A, MCA occlusion/reperfusion significantly up-regulated the expression of TNF-α, iNOS, IL-1β, COX-2 and IL-6 in the cerebral cortex compared with the non-ischemic side in the sham group. Treatment with GL significantly inhibited the expression of TNF-α, iNOS, IL-1β and IL-6 but had no apparent effect on the expression of COX-2. In contrast, co-treatment with rHMGB1 and GL again enhanced the expression of TNF-α, iNOS, IL-1β and IL-6 compared with the GL-pre-treatment alone group (P<0.01). The serum concentrations of TNF-α, iNOS, IL-1β and IL-6 in each group showed a similar trend to that of the mRNA expression levels, as determined by ELISA (Figure 4B). These results indicated that GL can inhibit the I/R-induced expression of HMGB1-dependent inflammation- and oxidative stress-related molecules in rat brains.

**Figure 5:**
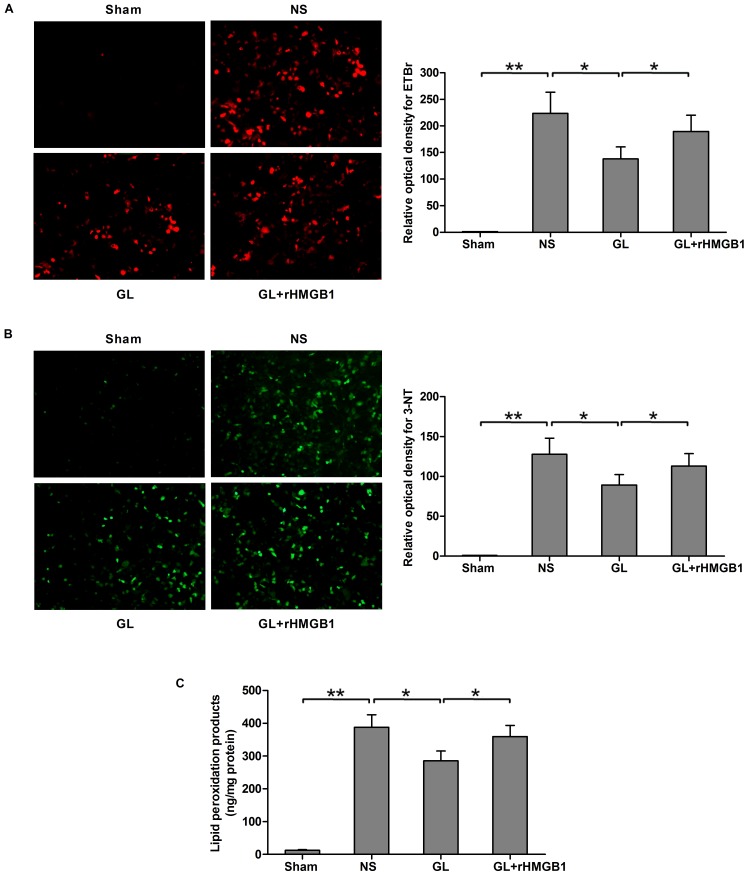
The antioxidant effects of GL are HMGB1 dependent. A) Fluorescent images after incubation with DHE showing superoxide levels in the I/R brain (red). Quantitative measurements of the fluorescence intensity indicated a significant change in the 4 mg/kg GL-treated group with or without rHMGB1, compared with the saline-treated I/R (NS) group. B) Representative photomicrographs showing 3-NT formation in the ischemic brain (green). Data from image analysis also indicated a significant change in the 4 mg/kg GL-treated group with or without rHMGB1, compared with the saline-treated I/R (NS) group. Bar = 50 µm. C) The effects of 4 mg/kg GL with or without rHMGB1 on MDA content in rat brains after I/R. Values are means±SEM, n = 8 for each group. *P<0.05, **P<0.01 (t test).

In the sham group, little DHE-induced EtBr fluorescence or 3-NT immunoreactivity was observed. The relative fluorescence intensities of superoxide-generating cells and 3-NT–positive cells were significantly increased in the NS-treated I/R hemisphere (Figure 5A–B). When rats were pre-treated with 4 mg/kg GL, the fluorescence intensity caused by superoxide and 3-NT production was significantly reduced compared with the NS group. In contrast, co-treatment with 4 mg/kg GL and 100 µg rHMGB1 enhanced the fluorescence intensity of superoxide and 3-NT production compared with the GL group, though the intensity was still lower than that in the NS group (Figure 5A–B). These histological results were further supported by a biochemical assay for MDA, a lipid peroxidation product. Pre-treatment with 4 mg/kg GL significantly decreased the level of MDA, compared with the NS group, while co-treatment with 100 µg rHMGB1 again increased the level of MDA, though this was still lower than that in the NS group (Figure 5C).

### GL modulates P38 and P-JNK but not p-ERK signalling

**Figure 6:**
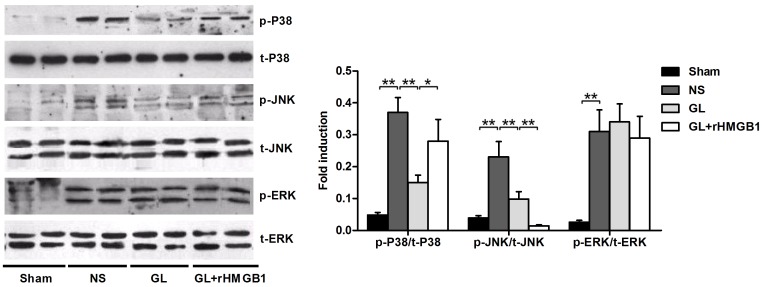
Representative blots showing the effects of GL with or without rHMGB1 treatment on phosphorylated (p) and total (t) p38, JNK, and ERK expression. Each blot shown is representative of 3 experiments with similar results. The bar graph showing semi-quantitative densitometric analysis summarises the fold change in phosphorylated to total p38, JNK and ERK in each group. Values are means±SEM, n = 8 for each group. *P<0.05, **P<0.01 (t test).

The mitochondrial-dependent apoptosis pathway is tightly regulated by the mitogen_activated protein (MAP) kinase family, and among these, JNK, ERK1/2, and p38 have been demonstrated to be activated during I/R injury [28], [29]. To study whether GL can modulate MAP kinase activity, we analysed these MAP kinases by immunoblotting. As shown in Figure 6, I/R induced phosphorylation of JNK, ERK, and p38 in isolated rat brain cerebral cortex. In GL-pre-treated rats, the phosphorylation levels of p38 and p-JNK were decreased compared with the NS-treated group, whereas p-ERK1/2 was not affected. Co-treatment with rHMGB1 enhanced the phosphorylation of p38 and JNK compared with the GL group, but the levels were still lower than those observed in the NS group. Pre-treatment with GL alone or with rHMGB1 did not affect the total levels of the JNK, ERK1/2, and p38 proteins. These results demonstrated that GL can modulate the p38 and JNK signalling pathways, but not the ERK signalling pathway, in the brains of MCA-occluded rats and that this effect is HMGB1 dependent.

## Discussion

Traditional Chinese medicine has become increasingly important in the treatment of cardiovascular ischemia/reperfusion injury. GL is a natural anti-inflammatory compound that is commonly used in Japan to treat patients with chronic hepatitis [14]–[17]. In the present study, we demonstrated that intravenous pre-treatment with a wide range of concentrations of GL, from 2 mg/kg to 10 mg/kg, reduced cerebral infarct volumes and ameliorated the neurological deficits of rat cerebral I/R injury. The use of a medium concentration of 4 mg/kg yielded considerable improvements, similar to those found at the high concentration of 10 mg/kg GL. Furthermore, this neuroprotective effect was accompanied by a reduction in the release of HMGB1 into the extracellular space and in the related inflammatory factors TNF-α, iNOS, IL-1β, and IL-6, both in the brain and serum of rats (Figure 2 and 4). Using this dose, no significant toxicities were observed in the effects investigated, including changes in the baseline hemodynamic values, cardiac electrophysiology, and in histopathological analysis of vital organs including liver, spleen, lung, kidney, and brain (data not shown).

HMGB1 is a nonhistone DNA-binding protein that possesses two HMG boxes that are DNA binding domains [30]. As a chromosomal protein, it has been implicated in diverse intracellular functions including stabilising nucleosomal structure and facilitating of gene transcription [31]. Moreover, HMGB1 is massively released extracellularly and plays a cytokine-like function in the post-ischemic brain [26], [32]. HMGB1 is actively secreted by macrophages and monocytes or released by necrotic cells into the extracellular milieu, where it might be involved in triggering inflammation [33]–[36]. The present study demonstrated that the predominantly nuclear HMGB1 staining of neurons in rats from the sham group weakened and disappeared during cerebral ischemia (Figure 2D). Thus, release from necrotic neural cells is the most likely source of elevated HMGB1 concentrations in the serum, which were further determined by immunoblot analysis in rats subjected to focal cerebral ischemia. A recent report of elevated serum concentrations of HMGB1 in stroke patients confirmed our findings [37]. Antagonising the pro-inflammatory function or blocking the expression of HMGB1 by a neutralising antibody [11], [12], HMGB1 box A [11], or a short hairpin RNA [10], [13] ameliorated brain damage. In particular, the expression of TNF-α, iNOS and IL-1β, which were all up-regulated in the post-ischemic brain, was reduced when HMGB1 was inhibited [10], [11], consistent with our results. HMGB1 has been reported to stimulate the production of IL-1, TNF-α, IL-6, and IL-8 and to induce iNOS expression [38], [39]. In the present study, we demonstrated that the ischemia-induced up-regulation of iNOS, IL-1β, IL-6 and TNF-α was inhibited by treatment with GL. Co-treatment with rHMGB1 can partially reverse this effect (Figure 4). The induction of iNOS and TNF-α following an ischemic insult was reported to occur mainly in microglia. Thus, it is likely that HMGB1 activates microglia in the brain, leading to the up-regulation of iNOS and TNF-α expression. In fact, the induction of iNOS and TNF-α has been reported to be involved in the inflammatory response and the disruption of the blood-brain barrier, leading to the aggravation of brain infarction [40], [41]. The regulation of any one of these factors has been postulated to reduce ischemic injury. Therefore, it is reasonable that GL, which has the ability to substantially reduce the expression of pro-inflammatory factors, exerted profound therapeutic effects on brain infarction.

In this study, pre-treatment with GL alleviated apoptosis injury resulting from cerebral I/R, and this was at least partly due to the inhibition of cytochrome C release and caspase 3 activity. Similarly, GL exhibited an anti-apoptotic effect by preventing HMGB1-induced cytochrome C release and caspase 3 activation in vitro in Huh-BAT cells [42]. Though the anti-apoptotic effect of GL has been linked with HMGB1 inhibition and caspase-dependent cytochrome c release, the question remains how GL regulates caspase-dependent cytochrome c release through the inhibition of HMGB1. The exact answer is not known at the present time, but we speculate that GL may modulate the activity of a particular kinase that contributes to cytochrome c translocation and is involved in I/R-induced HMGB1-dependent apoptosis.

Apoptosis is tightly regulated by the mitogen-activated protein (MAP) kinase family, and the JNK, ERK1/2, and p38 members of this family have been demonstrated to be activated in I/R injury. MAPKs play important roles in transducing signals by phosphorylating intracellular enzymes, transcription factors and cytosolic proteins involved in apoptosis and inflammatory cytokine production. Sustained MAPK activation has been shown to be associated with neuronal cell death/apoptosis following ischemic stroke, and the inhibition of this pathway is neuroprotective. In a Huh-BAT cell model, GL prevented HMGB1_induced cytochrome c release and p38 activation but had no effect on phospho_JNK and ERK1/2 [42]. In I/R-induced myocardial injury of rat heart, treatment with HMGB1 box A significantly reduced ERK1/2 and JNK phosphorylation, but did not affect the level of phospho-p38 [43]. However, in another similar study, treatment with GL significantly decreased JNK phosphorylation, but did not affect the level of phospho-p38 and ERK1/2^21^. Interestingly, in our studies, GL could modulate the p38 and JNK signalling pathways, but did not affect the ERK signalling pathway in the brains of MCA-occluded rats, and rHMGB1 could reverse this effect (Figure 6). This ambiguity regarding which single class of MAPK can be modulated by GL appears to primarily depend on the cell and tissue types used and differences in MAPK levels in vivo and in vitro.

In conclusion, our results demonstrated that pre-treatment with GL blocked and inhibited the extracellular cytokine activity of HMGB1 and explored the protective effect on I/R-induced apoptosis through the blockage of the JNK and p38-mediated pathways in rats in vivo. These data suggest a new therapeutic possibility for the treatment of ischemic stroke with GL. Considering the biological differences between species that may influence drug adsorption, metabolism, distribution, and toxicity in rats and humans, it is not clear whether GL has similar protective effects in humans. Hence, future research should be performed to evaluate the beneficial protective effect of GL in clinical settings with humans, as this might ultimately lead to a new therapeutic strategy for ischemic stroke.
